# A Capacitive Pressure Sensor with a Hierarchical Microporous Scaffold Prepared by Melt Near-Field Electro-Writing

**DOI:** 10.3390/s25092814

**Published:** 2025-04-29

**Authors:** Zhong Zheng, Yifan Pan, Hao Huang

**Affiliations:** Hubei Key Laboratory of Modern Manufacturing Quantity Engineering, School of Mechanical Engineering, Hubei University of Technology, Wuhan 430068, China; 102200023@hbut.edu.cn (Y.P.); 2010100508@hbut.edu.cn (H.H.)

**Keywords:** melt near-field electro-writing, hierarchical microporous, capacitive pressure sensor, MWCNT/PCL dielectric composites

## Abstract

Flexible capacitive pressure sensors (CPSs) have been widely studied and applied due to their various advantages. Numerous studies have been carried out on improving their electromechanical sensing properties through microporous structures. However, it is challenging to effectively control these structures. In this work, we controllably fabricate a hierarchical microporous capacitive pressure sensor (HMCPS) using melt near-field electro-writing technology. Thanks to the hierarchical microporous sensor, which provides a multi-level elastic modulus and relative dielectric constants, the HMCPS shows outstanding sensing properties. Its multi-range pressure response is sensitive: 3.127 kPa^−1^ at low pressure, 0.124 kPa^−1^ at medium pressure, and 0.025 kPa^−1^ at high pressure. Also, it has a stability of over 5000 cycles and a response time of less than 100 ms. The HMCPS can monitor dynamic and static pressures across a broad pressure range. It has been successfully applied to monitor human motions, showing great potential in human–computer interaction and smart wearable devices.

## 1. Introduction

Flexible pressure sensors [1] (FPSs) have been illustrated to have broad application prospects in fields such as human–computer interaction [2], wearable devices [3], and medical assistance [4], with the progress of technologies such as sensors, Internet of Things, and mobile computing [5,6,7]. FPSs can be divided into capacitive [8], piezoresistive [9], piezoelectric [10], triboelectric [11], and photoelectric [12] types, etc., according to the sensing mechanism. Among them, capacitive flexible pressure sensors [13,14,15] (CPSs) have been widely studied [16,17,18] because of their simple structure, low energy consumption, high resolution, fast response speed, and ease of large-area manufacturing. People are committed to developing CPSs with high sensitivity, low detection limits, and good linearity over a wide pressure range [19,20,21].

The capacitance of CPSs is defined as follows:

(1) $$C=\frac{\epsilon_{0}\epsilon_{r}A}{d}$$

Among these terms, ε_r_ is the relative permittivity of different dielectric materials, ε_0_ is the permittivity of vacuum, *A* is the relative area of the electrodes, and *d* is the distance between the upper and lower electrodes. *C* is a measure of the system’s ability to store electric charge. According to the definition, the change in capacitance in CPSs depends on the relative dielectric constant *ε_r_* of the dielectric materials, the distance *d* between the upper and lower electrodes, and the relative area *A* [22]. There are currently two mainstream solutions for developing the dielectric layer in order to improve the sensitivity of CPSs. One scheme is to introduce micro-structures with a low elastic modulus to make the dielectric layer highly compressible and thus improve sensitivity [23], such as microporous structures [24], micropillar structures [25], wrinkled structures [26], and foam structures [27], etc. Another scheme involves squeezing air with a low dielectric constant out of the pores of the dielectric layer during the compression process, replacing it with dielectric material that has a higher dielectric constant. The increase in the dielectric constant effectively improves the sensitivity of CPSs [28]. The way to obtain dielectric materials with a high dielectric constant is usually by mixing conductive fillers with a high dielectric constant in the polymer matrix, such as Carbon Nanotubes (CNTs) [29] and Ti_3_C_2_T_x_ MXenes [30]. The dielectric constant of polycaprolactone (PCL) is inherently low (approximately 2–3). However, the dielectric constant of the composite can be significantly increased (up to 10–100+) by adding MWCNTs into PCL, thereby enhancing the sensitivity of capacitive sensors.

The two aforementioned methods primarily demonstrate significant performance within a relatively narrow low-pressure range. However, under high-pressure conditions, the sensitivity of CPS typically declines sharply, leading to poor linearity and an inability to maintain high sensitivity across a broad detection range. This limitation hinders the practical application of CPSs in diverse scenarios [31]. Inspired by the structure of human skin, some hierarchical microporous micro-structures have been developed [32]. These structures achieve a gradient in the elastic modulus through variations in pore density. Regions with low pore density (and thus a low elastic modulus) are more responsive to low-pressure stimuli, while regions with high pore density (and a higher elastic modulus) are more responsive to high-pressure stimuli. Such stress self-adaptive characteristics significantly enhance compressibility and broaden the pressure response range of the structure. However, the currently reported fabrication techniques for hierarchical microporous structures—such as templating [33], etching [34], and electrodeposition [35]—suffer from a common limitation: they lack precise control over the shape, size, and spatial distribution of the micropores.

However, the currently reported preparation methods for hierarchical microporous structures include physical templating, chemical templating, and self-templating. Physical templating often utilizes porous materials with a defined pore structure as templates. For example, nanoporous silica can be used to create microporous structures in other materials through infiltration followed by removal of the silica template. Chemical templating involves the use of specific chemical agents that can guide the formation of micropores during synthesis. Self-templating, on the other hand, relies on the intrinsic structure or components of the material itself to generate the hierarchical microporous architecture. Each of these approaches offers distinct advantages and limitations, and researchers are actively exploring strategies to optimize and integrate them to achieve more precise control over micropore characteristics and improve the performance of materials with hierarchical microporous structures in various applications including catalysis, gas storage, and separation. Melt near-field electro-writing (MEW) [36] is an additive manufacturing technology for fabricate (sub)micron-scale fibers and complex structures. Compared with the traditional electrospinning technology, MEW enables the ordered and controllable deposition of fibers by shortening the spinning distance and coordinating the relative motion between the nozzle and the collector plate. Three-dimensional (3D) printing constructs objects by stacking materials layer by layer. One such technique, Fused Deposition Modeling (FDM), extrudes molten material layer by layer, which then solidifies, and finally forms a solid object. It is cost-effective but typically suffers from relatively low resolution. Compared with traditional 3D printing additive manufacturing technology, MEW can produce fibers at the micron scale—two orders of magnitude finer [37]. Most of the materials used in MEW are linear, hydrophobic, and non-cross-linked polymers. Among them, polycaprolactone (PCL) is the most common. Due to its good processing properties, biocompatibility, and degradability, it has broad application prospects in fields such as spun fibers, biomedical scaffolds, and drug delivery systems.

Here, we fabricated a CPS featuring a hierarchical microporous dielectric layer (HMCPS) using melt near-field electrospinning technology, enabling precise control over the microporous structure. The dielectric layer, composed of multiwalled carbon nanotube (MWCNT)/polycaprolactone (PCL) composites, exhibits a four-level graded microporous architecture with decreasing pore size from top to bottom. The HMCPS demonstrates a sensitivity of 3.127 kPa^−1^ in the low-pressure range (50–15 kPa), 0.124 kPa^−1^ in the mid-pressure range (15–100 kPa), and 0.025 kPa^−1^ in the high-pressure range (100–400 kPa). This performance surpasses that of previously reported capacitive pressure sensors employing porous media layers or porous electrodes in terms of both sensitivity and sensing range. Moreover, the HMCPS exhibits excellent durability over 5000 loading cycles and a rapid response time (<100 ms). As a proof of concept, the sensor effectively monitored physiological signals such as joint and muscle movements and pulse waves, highlighting its potential in human–machine interaction and wearable intelligent devices.

## 2. Materials and Methods

### 2.1. Materials

Multi-walled carbon nanotubes (MWCNTs) were obtained from Zhongke Shidai Nanomaterials Co., Ltd. (Beijing, China). Polycaprolactone (PCL) was acquired from Perstrop CAPA6400 in Malmö, Sweden. Lamellar ternary carbide Ti_3_AlC_2_ powder (MAX phase, 200 mesh) was provided by Jilin Shiyi Technology Company (Jilin, China). 3M Tegaderm was obtained from 3M Medical Devices Co., Ltd. Polyimide (PI) tape was sourced from Shanghai Mingshen Electronic Technology Development Co., Ltd. (Shanghai, China). Carbon nanotube water-dispersing agent was acquired from Nanjing Xianfeng Nanomaterials Technology (Nanjing, China). Lithium fluoride (LiF, AR, 98%), concentrated hydrochloric acid (36–38%), and anhydrous ethanol were provided by Sinopharm Chemical Reagent Co., Ltd. (Wuhan, China). Deionized water was used in all experiments.

### 2.2. Preparation of MWCNTs/PCL Dielectric Layer

**Figure 1:**
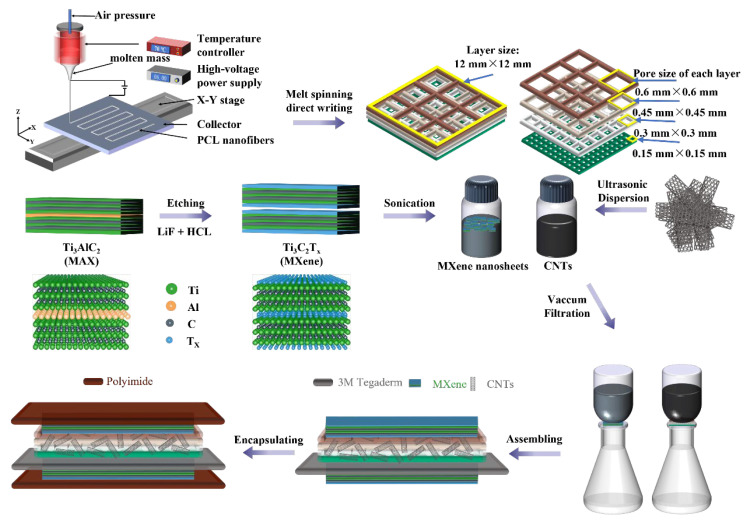
Schematic diagram of HMCPS preparation process.

A schematic diagram of the HMCPS preparation process is illustrated in Figure 1. Firstly, a PCL scaffold with a hierarchical microporous structure was prepared through melt near-field electro-writing. The raw material for melt electrospinning direct writing was solid PCL particles that had been vacuum-dried (40 °C, 12 h). Various parameters were set (melting temperature: 70 °C, voltage: 6 kV, air pressure: 30 kPa, needle tip height: 5 mm, number of electrospinning layers: 16 layers, room temperature: 22–30 °C, humidity: about 40%, size of each layer: 12 × 12 mm, collector speed: 20 mm/s, and collector acceleration: 3 mm/s^2^). Three PCL layer scaffolds with different pore densities were electrospun layer by layer, respectively: (1) 16-layer square equal-sized pores with a side length of 0.15 mm as a high-density pore structure; (2) 16-layer square equal-sized pores with a side length of 0.6 mm as a low-density pore structure; (3) every 4 layers of square equal-sized pores with different side lengths were regarded as one level, and the side lengths of the 4 levels from bottom to top were 0.15 mm, 0.3 mm, 0.45 mm, and 0.6 mm in sequence. Then, MWCNTs were dispersed in deionized water, and the aqueous dispersant with a MWCNT content of 30% was dropped in and ultrasonicated intermittently for 30 min in a probe-type ultrasonic instrument to obtain a uniformly dispersed MWCNT suspension. Finally, MWCNTs were deposited on the PCL layer scaffold by vacuum filtration and vacuum-dried at 40 °C for 24 h to obtain a hierarchical microporous MWCNT/PCL composite as a dielectric layer.

### 2.3. Preparation of MXene Electrode Layer

The Ti_3_C_2_T_x_ MXene aqueous dispersion was prepared by the LiF and HCl liquid-phase etching method. Firstly, 2 g of LiF was dissolved in 40 mL of 9 M HCl and stirred at room temperature for 30 min to make the system fully react. Then, Ti_3_AlC_2_ powder (2 g) was slowly added to the mixed solution of HCl and LiF and magnetically stirred in a PTFE reactor at 35 °C for 24 h (400 rpm) to ensure sufficient etching of Al. The obtained suspension was divided into four centrifuge tubes and washed several times by centrifugation with deionized water at 3500 rpm (8 min, 20 mL deionized water) until the pH value increased to 6. The washed precipitate was dispersed in 40 mL of deionized water, and then ultrasonicated in an ice bath for 1 h for further stratification. The supernatant was obtained by centrifuging the suspension at 3500 rpm for 30 min to obtain a single-layer and few-layer MXene solution. Finally, MXene was deposited on an aqueous-phase microporous filter membrane with a pore size of 0.22 μm using vacuum-assisted filtration, vacuum-dried at 60 °C for 24 h to obtain an independent MXene film with a thickness of about 100 μm, and cut into the size required for the electrode.

### 2.4. Assembly of HMCPS

**Figure 2:**
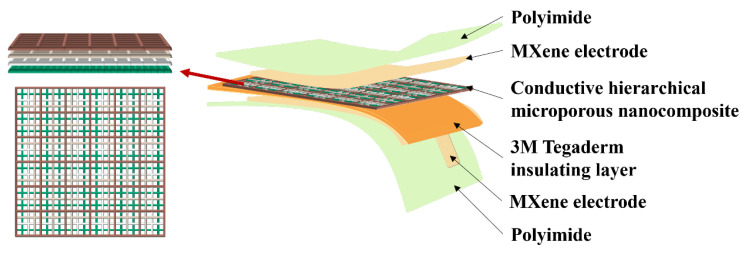
Exploded schematic illustration of a HMCPS.

The electrode layer MXene was cut into the same length and width as the MWCNT/PCL hierarchical microporous dielectric layer. As illustrated in the exploded illustration in Figure 2, the dielectric layer and the piezoresistive layer are MWCNT/PCL conductive hierarchical microporous nanocomposite layers, the MXene electrode layers are located above and below them, and the 3M Tegaderm insulating layer with a thickness of 20 μm is sandwiched between the lower electrode and the composite layer to prevent short circuiting and CNT leakage. Finally, the above-mentioned multilayers were encapsulated with two transparent ultra-thin PI tapes with a thickness of 500 nm to isolate the interference of external environmental signals and fix the relative positions of the upper and lower electrodes and other layers at the same time.

### 2.5. Characterization and Measurements:

The dielectric layer structure was detected using scanning electron microscope (SEM, Mira, Tescan, Brno, Czech Republic). The most important parts in the sensor performance test are the pressure-applying and signal-collecting devices. The dynamic pressure was controlled and measured with a universal testing machine (CMT4204, Mitter Industrial Systems Co., Ltd., Shanghai, China), and the corresponding capacitance change was measured with an LCR meter (TH2832, Tonghui Co., Ltd., Changzhou, China). The sensor sample was fixed on the sample platform of the universal testing machine and connected to the LCR meter with wires. A computer was placed to set the pressure and record the change in capacitance value. The physical pictures of the specific experimental devices are given in Supporting Information Figure S1.

Experiments involving human subjects: Experiments involving human subjects were carried out with full informed consent from the volunteers. All reported tests were in line with the ethical requirements of the Hubei University of Technology.

## 3. Results

### 3.1. Structure and Morphology

**Figure 3:**
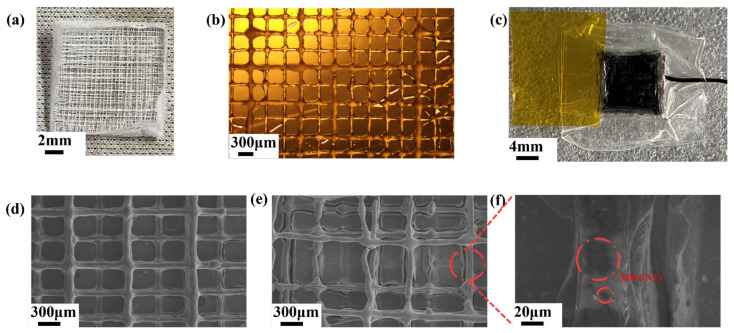
Upper surface of the hierarchical microporous structures. Optical photographs of (**a**) PCL scaffold. (**b**) PCL scaffold with a pore size of 0.3 mm × 0.3 mm. (**c**) Hierarchical microporous MWCNT/PCL composite. SEM diagram of (**d**) MWCNT/PCL composite with a pore size of 0.3 mm × 0.3 mm. (**e**,**f**) Hierarchical microporous MWCNT/PCL composite.

The core of the HMCPS comprises a hierarchical microporous MWCNT/PCL composite as the sensitive layer. The hierarchical microporous structures of the PCL scaffold and MWCNT/PCL composite are illustrated in Figure 3. The diameter of a single fiber produced by MEW is basically between 40 and 60 μm. Figure 3d shows the microscopic morphology of the monopole isoporous structure with a pore size of 0.3 mm. A total of 16 layers were continuously spun, and the position of each layer was fixed, providing certain support when spinning the next layer. Therefore, each fiber was very regular and orderly. The micro-morphologies of the other three pore sizes (0.15 mm, 0.45 mm, and 0.6 mm) are similar to this and will not be elaborated here anymore. Figure 3e shows an SEM (scanning electron microscope) image of the micro-morphology of the hierarchical microporous structure. There are four levels of different pore sizes from bottom to top, and four layers are spun at each level. It can be seen that some fibers at the top level show breakpoints, or their positions are shifted. This is mainly because, due to the different pore sizes at each level, some fibers will lose the support of the next level, so they will sink into the next level or be truncated at the intersection of the next level. On the other hand, as the number of spun layers increases, the electrostatic force between the fibers affects the continuous orderly and accurate deposition of the fibers. Sixteen layers are the current limit of the hierarchical pores in the experiment, so some fibers are inclined. Figure 3f is a partially enlarged view of Figure 3e, which shows the deposition situation on the surface of MWCNT (multi–walled carbon nanotube) fibers, and MWCNTs are marked with red circles. In addition, in order to clearly characterize the hierarchical microporous structure further, the SEM image of cross-section scanning is illustrated in Figure S2 of the Supporting Information.

### 3.2. Electromechanical Performance

**Figure 4:**
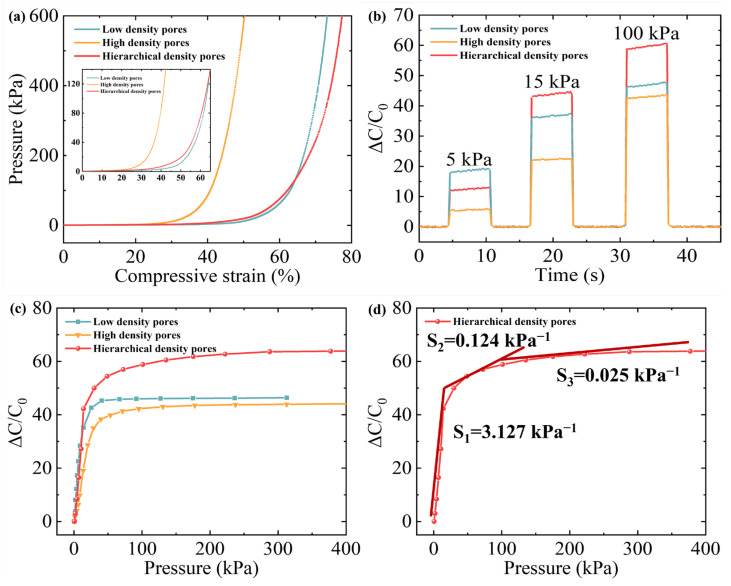
Performance of CPSs made of different pore structures. (**a**) Pressure–strain curves. (**b**) Relative capacitance variation at different pressures. (**c**) Relative capacitance curves under pressure response. (**d**) Pressure–relative capacitance response of HMCPS.

To systematically evaluate the influence of pore structure on sensor performance, three distinct polycaprolactone (PCL) layer scaffolds were fabricated via melt electro-writing (MEW) for comparative analysis: a high-density pore structure with equally small square pores (0.15 mm × 0.15 mm) from bottom to top, a low-density pore structure with equally large pores (0.6 mm × 0.6 mm), and the hierarchical density pores in our HMCPS, decreasing successively from top to bottom (0.6 mm × 0.6 mm, 0.45 mm × 0.45 mm, 0.3 mm × 0.3 mm, and 0.15 mm × 0.15 mm). These scaffolds served as dielectric layer substrates to construct three capacitive pressure sensors (CPSs). The mechanical and electrical responses of each CPS variant were characterized, as detailed in Figure 4. Five samples of each structure were tested. There was little difference in performance among the samples, and sufficient capacitance stability was achieved. From Figure 4a, the hierarchical microporous structure (HMCPS) has significantly larger compressive strain than the high-density pore structure under the same pressure and can accept larger pressure sensing than the low-density pore structure. After connecting to the digital bridge, the relative capacitance changes in CPSs made of three different pore structures under different pressures were tested, respectively, as illustrated in Figure 4b, and the relative capacitance curves under continuous pressure responses are illustrated in Figure 4c. The initial capacitance is 2 pF. The results show that the relative capacitance response of the HMCPS under low pressure is slightly inferior to that of the CPS made of low-density pores. The reasons for this phenomenon will be elaborated upon specifically in the mechanism analysis in the next section. The HMCPS has excellent capacitance response performance and a wide pressure sensing range under moderate and high pressures, which proves that the hierarchical microporous structure medium shows a superior relative capacitance response in a wide pressure range. Specifically, the relative capacitance response of the HMCPS with 0.2 wt% MWCNT loading in a wide pressure range is illustrated in Figure 4d. Benefiting from the hierarchical microporous structure, the highly sensitive linear response part of the HMCPS can be as high as 15 kPa. The sensitivity of the HMCPS is 3.127 kPa^−1^ within 15 kPa, 0.124 kPa^−1^ within 15–100 kPa, and 0.025 kPa^−1^ within 100–400 kPa. Compared to the other two microporous structures, it has a more obvious three-stage linear sensitivity. Although the linear sensitivity in the case of low-density pores under low pressure is slightly higher, the HMCPS has a wider linear response range, and its sensitivity under high pressure is much higher than that of the other two structures.

### 3.3. Mechanism Analysis

Then, a mechanism analysis was carried out on the HMCPS to obtain a relatively high sensing performance. As an important parameter for evaluating the performance of a sensor, sensitivity S is defined as follows:

(2) $$S=\frac{\delta (\Delta C/C_{0})}{\delta P}$$

Among these terms, $\Delta C/C_{0}$ is the relative capacitance variation, which is derived from the ratio of the capacitance variation value ($\Delta C=C-C_{0}$) to the initial capacitance ($C_{0}$). P is the applied pressure.

The initial capacitance $C_{0}$ can be described as follows:

(3) $$C_{0}=\frac{\epsilon_{0}\epsilon_{r0}A}{d_{0}}$$

The calculation formula for the elastic modulus E is the following:

(4) $$E=\frac{P}{\left(\Delta d/d_{0}\right)}$$

Therefore, sensitivity can be defined as follows:

(5) $$S=\frac{\Delta C/C_{0}}{P}=\frac{\left(\epsilon_{0}\epsilon_{r}A/d-\epsilon_{0}\epsilon_{r0}A/d_{0}\right)}{\left(\epsilon_{0}\epsilon_{r0}A/d_{0})(E\Delta d/d_{0}\right)}=\frac{d_{0}}{Ed}(1+\frac{\Delta \epsilon /\epsilon_{r0}}{\Delta d/d_{0}})$$

**Figure 5:**
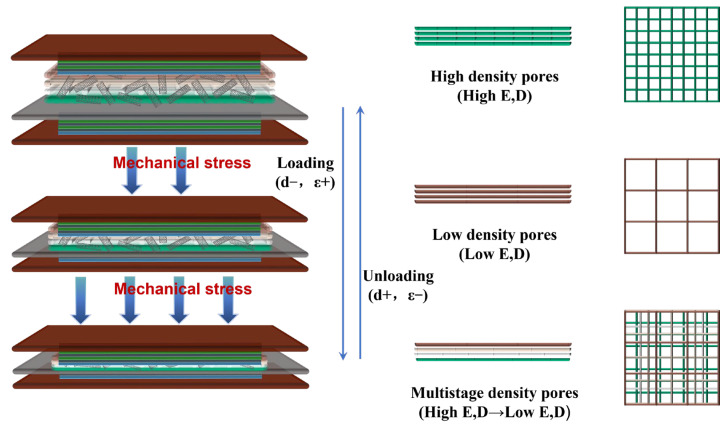
Schematic illustration of the sensing mechanism for HMCPS.

In this formula $\Delta \epsilon =\epsilon_{r}-\epsilon_{r0}$, it can be inferred that sensitivity mainly depends on the ratio of $\Delta \epsilon /\epsilon_{r0}$ and $\Delta d/d_{0}$. As illustrated in Figure 5, due to the pore structure, the distance between the two electrodes can easily become $d_{0}-\Delta d$ under normal pressure. Media with different pore densities have different elastic moduli under pressure load. The low-density porous structure exhibits a reduced elastic modulus, enabling significant deformation under minimal pressure, which enhances its sensitivity to low-pressure stimuli. However, this architecture is prone to premature saturation during compression due to excessive deformation, consequently limiting its operational pressure range. In contrast, the high-density porous structure demonstrates an elevated elastic modulus and consequently exhibits limited compressibility under low-pressure conditions. At this time, the change in $\Delta d/d_{0}$ is very small and the sensitivity is low. Therefore, a PCL medium with hierarchical pore density was prepared using melt-spinning direct writing technology. The hierarchical elastic moduli increase successively from top to bottom, meeting the requirements for high sensitivity under different pressures.

In order to obtain high sensitivity in a wider range, $\Delta \epsilon /\epsilon_{r0}$ is another key parameter. Studies have illustrated that adding conductive fillers to polymers can lead to a large dielectric constant. With the increase in the load, the MWCNT/PCL composite dielectric material with a high dielectric constant continuously replaces the air gaps with a low dielectric constant in the dielectric layer, resulting in an excellent relative capacitance variation. Specifically, the effective dielectric constant ($\epsilon_{e}$) of the dielectric layer can be expressed as follows:

(6) $$\epsilon_{e}=\epsilon_{0}\times \varphi_{0}+\epsilon_{MP}\times \varphi_{MP}$$

(7) $$\varphi_{0}+\varphi_{MP}=1$$

Among these terms, $\varphi_{0}$ is the volume fraction of the air gap (air), and $\varphi_{MP}$ is the volume fraction of the MWCNT/PCL composite layer. During the process of applying load, the volume fraction of the air gap continuously decreases, the volume fraction of MWCNT/PCL composite layer continuously increases, and the relative dielectric constant increases. By using the synergetic effect of Δϵ and Δd, the sensitivity of the capacitive sensor can be significantly enhanced through structural optimization. In this study, MWCNTs were deposited via vacuum filtration onto a PCL scaffold featuring a hierarchical density porous structure. The hierarchical architecture plays a critical role in performance improvement. Specifically, the upper low-density porous layer contains larger pores, resulting in a high initial air gap volume fraction. Under external pressure, these air gaps are progressively replaced, leading to a substantial change in relative capacitance and thereby achieving high sensitivity to minor stimuli. Furthermore, the MWCNT content exhibits a graded distribution, increasing from the top to the bottom layer. The high-density porous base layer, characterized by its elevated dielectric constant, maintains high sensitivity even under significant compressive pressures.

**Figure 6:**
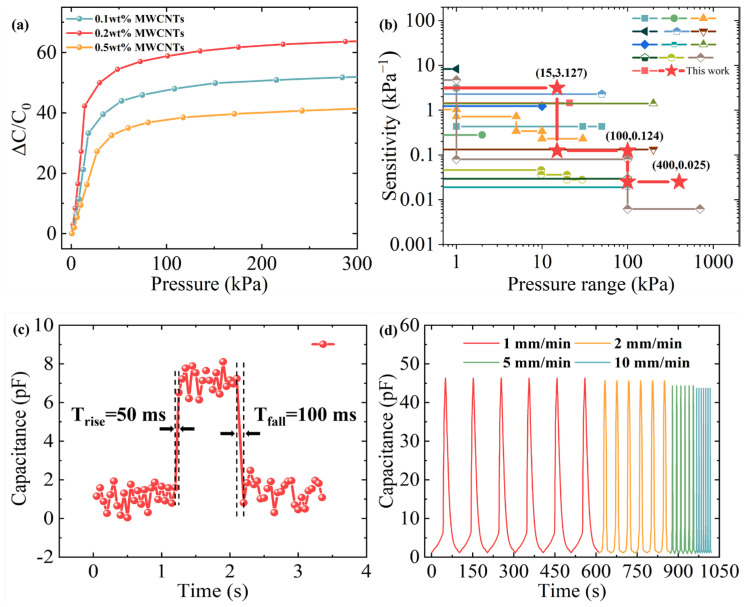
Electromechanical response of HMCPS. (**a**) Pressure–relative capacitance variation curve of HMCPS containing 0.1 wt%, 0.2%, and 0.5 wt% MWCNTs. (**b**) Comparison of HMCPS with state-of-the-art counterparts. (**c**) Response and recovery time test under a pressure load of 10 Pa. (**d**) Capacitance response curves during cyclic compression at different frequencies [1,2,3,4,6,8,14,15,18,19,21,30,31].

In order to study the influence of MWCNT loading on sensing performance, relevant tests were carried out on sensors made of hierarchical microporous structure media with different MWCNT loadings. It can be seen from Figure 6a that with the increase in MWCNT loading, the C_0_ of the HMCPS gradually increases, while $\Delta C/C_{0}$ first increases significantly and then begins to decrease, starting from 0.2 wt% of MWCNT loading. With the increase in MWCNT loading, the effective dielectric constant ($\Delta \epsilon /\epsilon_{r0}$) of the medium increases, which is helpful for improving sensitivity. However, the increase in excessive MWCNT loading will cause an increase in C_0_, affect the compressive modulus of the medium, and lead to an insignificant change or even decrease in the value of $\Delta C/C_{0}$.

**Figure 7:**
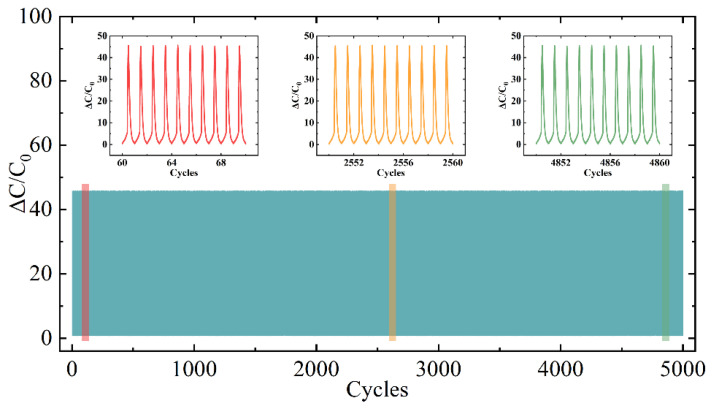
Long-term stability of HMCPS under 15 kPa for 5000 cycles.

Compared to conventional capacitive pressure sensors (Figure 6b), the hierarchical microporous capacitive pressure sensor (HMCPS) demonstrates superior performance in both sensitivity and the pressure detection range. Response time, a critical parameter for evaluating sensor dynamics, was systematically characterized. As shown in Figure 6c, the HMCPS exhibits rapid response to low-pressure stimuli (10 Pa), achieving a response time of 50 ± 10 ms and recovery time of 100 ± 15 ms. These response characteristics are significantly faster than typical human motion frequencies, indicating the device’s capability for high-frequency dynamic signal detection. In order to detect the stability of the HMCPS in practical application scenarios, 10 kPa pressure signals with different compression frequencies of 1, 2, 5, and 10 mm/min were applied to the sensor surface. The curve in Figure 6d shows that under the external pressure stimulation of different frequencies, the HMCPS can always give a stable signal response. In addition, in order to evaluate the repeatability and stability of the HMCPS under static and dynamic responses, a loading–unloading cyclic pressure of 15 kPa was applied at a compression frequency of 2 mm/min, and a total of 5000 cycles were carried out. As illustrated in Figure 7, from loading to unloading, the HMCPS achieved good repeatability and stability. Given that parasitic effects are a well-documented limitation of capacitive sensors, due to the low melting point of PCL, at high temperatures, the hierarchical microporous structure will be destroyed because the PCL layer scaffold will melt, so the sensor cannot maintain its original performance at high temperatures. At different humidity levels, as illustrated in Supporting Information Figure S3, the real-time performance of the sensor does not change much, demonstrating good humidity stability.

### 3.4. Application

**Figure 8:**
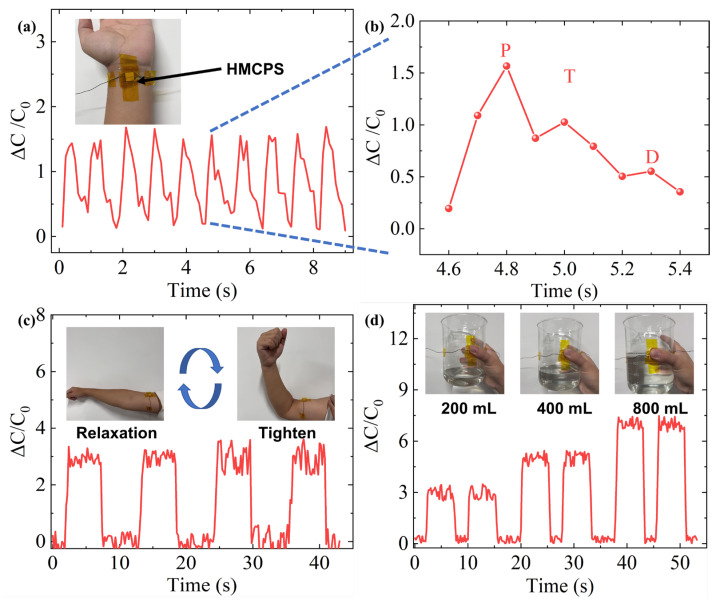
The application of HMCPS. (**a**) Pulse capacitance response. (**b**) Locally enlarged characteristic peaks. (**c**) Contraction and relaxation of biceps brachii. (**d**) Fingers grasping beakers of different masses.

The HMCPS demonstrates exceptional sensing performance across a broad pressure range. To systematically evaluate its potential for flexible wearable device applications, we conducted a series of experiments encompassing multiple scenarios including biosignal monitoring and motion detection. For cardiovascular monitoring, the HMCPS was affixed to the radial artery of a volunteer’s wrist to acquire real-time blood pressure waveforms. As illustrated in Figure 8a, the sensor successfully captured periodic arterial pulsations and generated continuous waveform profiles. Detailed analysis of the radial artery waveform (Figure 8b) revealed three distinct physiological components: (1) the percussion wave, corresponding to cardiac systole and reflecting myocardial contractility; (2) the tidal wave, associated with vascular elasticity and peripheral resistance during blood reflux; and (3) the diastolic wave, representing vascular compliance during cardiac diastole. This high-fidelity signal acquisition capability underscores the sensor’s exceptional sensitivity to subtle hemodynamic changes, demonstrating potential for clinical applications such as arteriosclerosis assessment and cardiac function evaluation.

For musculoskeletal monitoring applications, the HMCPS was positioned on the biceps brachii with optimal skin conformality (Figure 8c). The sensor exhibited rapid response to both muscle contraction (increased capacitive signal) and relaxation (pressure decay), enabling the continuous monitoring of dynamic biomechanical states. This performance suggests promising applications in sports science (e.g., training intensity quantification), rehabilitation medicine (e.g., recovery progress tracking), and human–machine interfaces.

Furthermore, we evaluated the sensor’s capability in grip analysis by mounting the HMCPS on laboratory beakers of varying capacities (200, 400, and 800 mL; Figure 8d). The system reliably discriminated pressure distributions associated with different loading conditions while providing real-time force monitoring. This functionality demonstrates considerable potential for studies in hand biomechanics, including muscle coordination analysis and grip strength optimization.

The aforementioned practical applications comprehensively validate the outstanding performance characteristics of the HMCPS. The sensor demonstrates: (1) an exceptionally broad pressure detection range (up to 400 kPa); (2) ultrahigh sensitivity (3.127 kPa^−1^); (3) rapid response dynamics (<100 ms); and (4) superior pressure resolution (10 Pa), coupled with minimal hysteresis, excellent repeatability (>10,000 cycles), and remarkable long-term stability (>30 days). Furthermore, the HMCPS exhibits exceptional wearability due to its ultralight weight (30 mg) and compact footprint (20 × 20 mm^2^), enabling imperceptible epidermal integration while maintaining excellent biocompatibility.

## 4. Conclusions

In summary, we report a high-performance flexible CPS with controllable fabrication, featuring a four-level microporous MWCNT/PCL composite dielectric layer. The structure offers a multi-level elastic modulus and relative dielectric constants, enabling superior sensing across a broad pressure range. The HMCPS shows sensitive responses in multiple pressure ranges (3.127 kPa^−1^ at 50–15 kPa, 0.124 kPa^−1^ at 15–100 kPa, and 0.025 kPa^−1^ at 100–400 kPa), along with excellent stability (over 5000 cycles) and rapid response time (<100 ms). The successful use of the HMCPS in monitoring human movements, such as joint motion and pulse beats, proves its real-world potential. Its ability to detect dynamic and static pressures across a wide range makes it suitable for various wearable and interactive applications. MEW technology enables precise control over the microporous structure, crucial for sensor performance optimization and application reliability. Besides the four-level microporous structure in this study, micropores of diverse shapes and sizes can also be manufactured. Future work can focus on further enhancing sensor performance, exploring new materials and structures, and expanding its applications in different fields.

## Data Availability

Data are contained within the article.

## Supplementary Materials

The following supporting information can be downloaded at: https://www.mdpi.com/article/10.3390/s25092814/s1, Figure S1: diagram of characterization and testing experimental devices. (a) Universal mechanical testing machine of type CMT4204. (b) Hitachi SU8010 scanning electron microscope.; Figure S2: SEM cross-sectional view of hierarchical microporous structure MWCNT/PCL conductive composite layer.; Figure S3: The real-time capacitance response of HMCPS at 15 kPa in different humidity environments. References [38,39] are cited in Supplementary Materials.
