# Supplementary material for: A Capacitive Pressure Sensor with a Hierarchical Microporous Scaffold Prepared by Melt Near-Field Electro-Writing
Source: Sensors (Basel). 2025 Apr 29;25(9):2814. doi: 10.3390/s25092814 (PMC12074285; doi:10.3390/s25092814)
Supplement: Supplementary file 1 [file sensors-25-02814-s001.zip › sensors-3582416-supplementary.pdf]

Supporting Information

A Capacitive Pressure Sensor with Hierarchical Microporous Scaffold Prepared by Melting Near-field Electro-writing

Zhong Zheng[-1],*，Yifan Pan[-1]，Hao Huang[-1]

1 Hubei Key Laboratory of Modern Manufacturing Quantity Engineering, School of Mechanical Engineering, Hubei University of Technology, Wuhan, Hubei 430068, China;102200023@hbut.edu.cn (Y.P.); 2010100508@hbut.edu.cn (H.H.)

* Correspondence: zhengzh@hbut.edu.cn (Z.Z.); Tel: +86-027-59750012 (Z.Z.)

1. The reasons for choosing MWCNTs/PCL combination.

The dielectric constant of poly(caprolactone) (PCL) is inherently low (approximately 2 - 3)[1]. However, the dielectric constant of the composite can be significantly increased (up to 10 - 100+) by adding MWCNTs into PCL, thereby enhancing the sensitivity of capacitive sensors[2]. The conductive network of MWCNTs forms a micro-capacitor structure in the PCL matrix. Changes in the conductive pathways induced by external forces directly modulate the capacitance response, thereby enabling high-sensitivity pressure detection. The low melting viscosity of PCL (60 - 80°C) and the good dispersion of MWCNTs in it facilitate the stable fabrication of micron-scale fibers via MEW. The process generates porous or grid-like structures, which enhance the compression resilience and response speed of the sensor.

Other combinations, such as using TPU instead of PCL. However, the melting point of TPU is too high and is greatly affected by environmental factors during the preparation process. These characteristics lead to the collapse of hierarchical microporous structures and nozzle blockages, thereby hindering the stable and rapid production of micron-level fibers. When graphene-based materials (e.g., graphene oxide (GO) or reduced graphene oxide (rGO)) were used instead of MWCNTs, GO tends to aggregate, preventing the formation of a stable hierarchical dielectric structure. Additionally, the reduction process for rGO is complex and may introduce toxicity,

further limiting its practical application.

2. Experimental device physical diagram

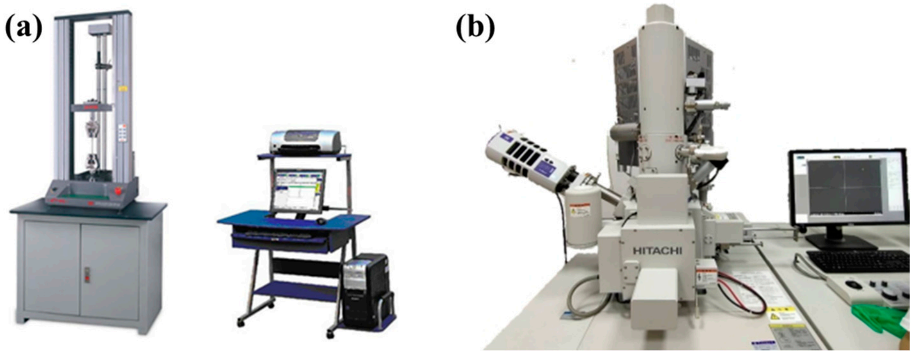

Figure S1. Diagram of Characterization and Testing Experimental Devices. (a) Universal mechanical testing machine of type CMT4204. (b) Hitachi SU8010 scanning electron microscope.

2. SEM cross section of MWCNTs / PCL composite layer with hierarchical microporous structure

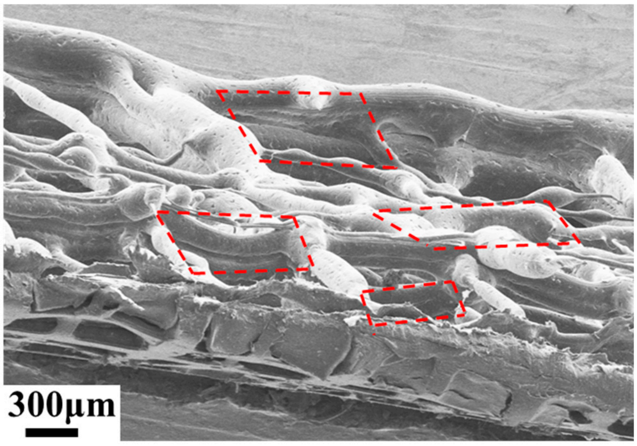

Figure S2. SEM cross - sectional view of hierarchical microporous structure MWCNTs/PCL conductive composite layer.

3. The influence of humidity environment on the sensing response of HMCPS

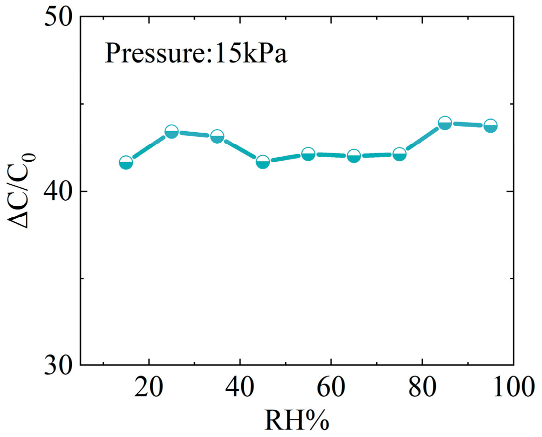

Figure S3 The real-time capacitance response of HMCPS at 15 kPa in different humidity

environments

References

1. Pawar, R.; Pathan, A.; Nagaraj, S.; Kapare, H.; Giram, P.; Wavhale, R., Polycaprolactone and its derivatives for drug delivery. *Polymers for advanced technologies* **2023,** 34, (10), 3296-3316.
2. Tu, J.; Chu, C.; Gao, Y.; Wang, Z.; Xu, P.; Ding, Y., Enhanced dielectric and mechanical properties of polylactic acid/polycaprolactone blends by introducing double - layer carbon nanofillers. *Journal of Applied Polymer Science* **2024,** 141, (5), e54874.
